# Primary Hepatic Lymphoma Is Difficult to Discriminate from a Liver Abscess

**DOI:** 10.1155/2014/925307

**Published:** 2014-03-16

**Authors:** Nobuhiro Takeuchi, Kazuyoshi Naba

**Affiliations:** ^1^Department of Gastroenterology, Kawasaki Hospital, Kobe, Hyogo 652-0042, Japan; ^2^Department of Laboratory Medicine, Kawasaki Hospital, Kobe, Hyogo 652-0042, Japan

## Abstract

An 82-year-old woman presented with a high-grade fever of 40°C and was admitted to our institution for intensive examination and treatment. Noncontrast abdominal computed tomography (CT) revealed low-density masses at segments 5 and 8, suggestive of a liver abscess. On further examination, a contrast-enhanced abdominal CT showed a 30 × 30 mm mass with an enhanced margin at segment 8 in the arterial phase; the contrast agents were washed out in the venous phase. In addition, a 63 × 52 mm mass with a density lower than that of liver parenchyma was observed at segment 8 in the portal phase. On the basis of these findings, either a liver abscess or hepatocellular carcinoma was suspected. To confirm the diagnosis, a fine needle biopsy was scheduled. Histopathological analysis of the biopsied specimens confirmed the diagnosis of diffuse large B-cell lymphoma. Chemotherapy was not indicated owing to the patient's age and poor performance status; thus, best supportive care was planned. On day 22 after admission, the patient died of pneumonia. We experienced a case of PHL that was difficult to discriminate from a liver abscess. Imaging alone is insufficient to diagnose PHL; therefore, fine needle biopsy is recommended for a definitive diagnosis.

## 1. Introduction

Primary hepatic lymphoma (PHL) is a rare entity comprising only 0.48% of all malignant lymphoma. PHL is sometimes difficult to diagnose accurately as there are no specific imaging characteristics associated with this disease and confirmation requires a fine needle biopsy. Here we present a case of PHL that was difficult to discriminate from a liver abscess; fine needle biopsy facilitated diagnosis confirmation.

## 2. Case Presentation

**Figure 1:**
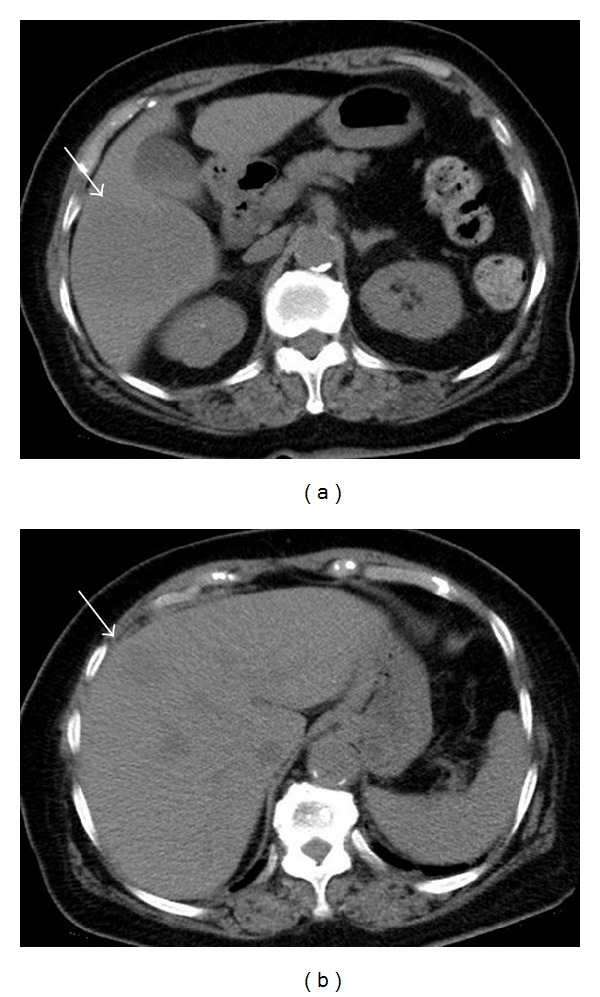
Noncontrast abdominal computed tomography. Low-density masses were revealed at segments 5 and 8, suggestive of a liver abscess ((a) and (b)).

**Figure 2:**
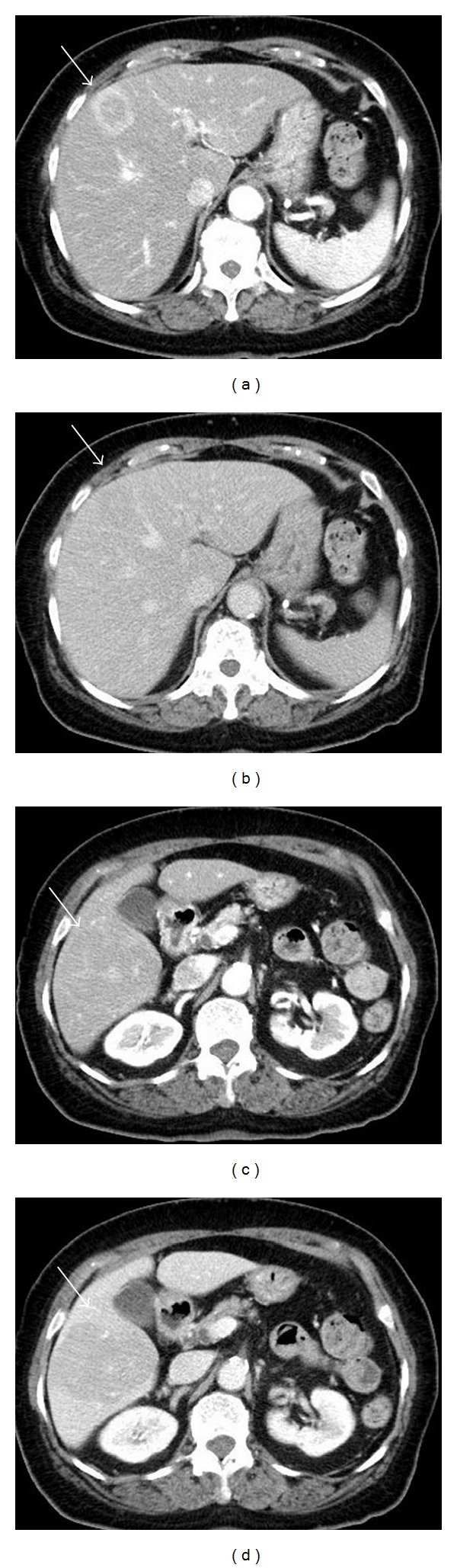
Contrast-enhanced abdominal computed tomography. A 30 × 30 mm mass with an enhanced margin was revealed at segment 8 in the arterial phase (a). The contrast agents were washed out in the venous phase (b). A 63 × 52 mm mass with a density lower than that of liver parenchyma was revealed at segment 8 in the portal phase (c). On the basis of these findings, either a liver abscess or hepatocellular carcinoma was suspected.

**Figure 3:**
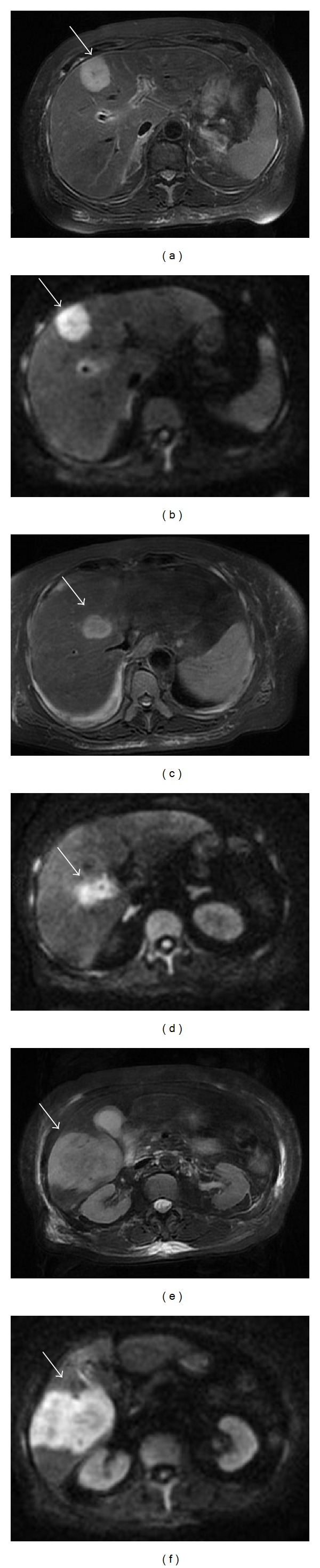
Noncontrast abdominal magnetic resonance imaging. Masses, 3.2 cm, 7 cm, and 2.2 cm in size, were revealed at segments 5, 6, and 8, respectively ((a)–(f)). These masses exhibited high signal intensity on T2- and diffusion-weighted imaging. Penetrating vessels were evident among the masses, suggesting hepatic lymphoma.

**Figure 4:**
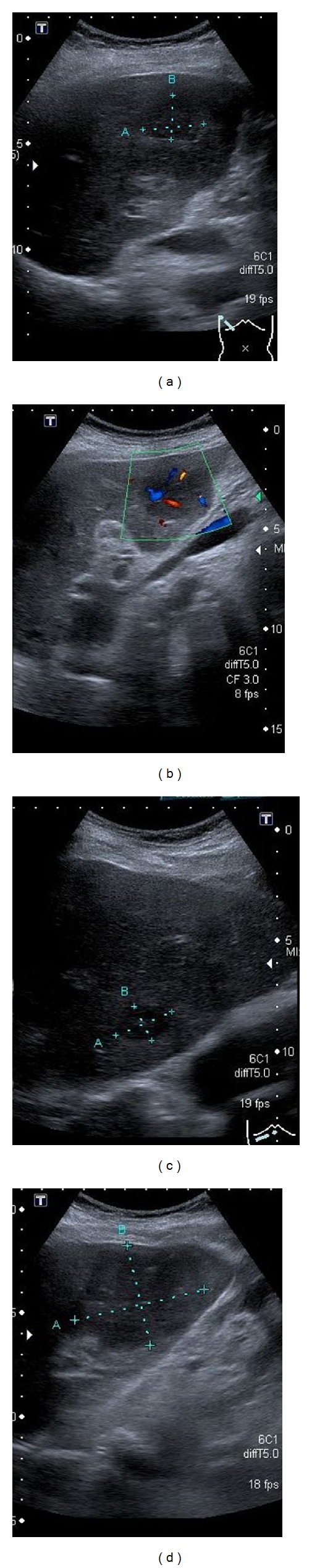
Ultrasonography. Low-echo masses, 3 cm, 6 cm, and 3 cm in size, were revealed at segments 5, 6, and 8, respectively ((a)–(d)). Penetrating vessels were evident among the masses (b).

**Figure 5:**
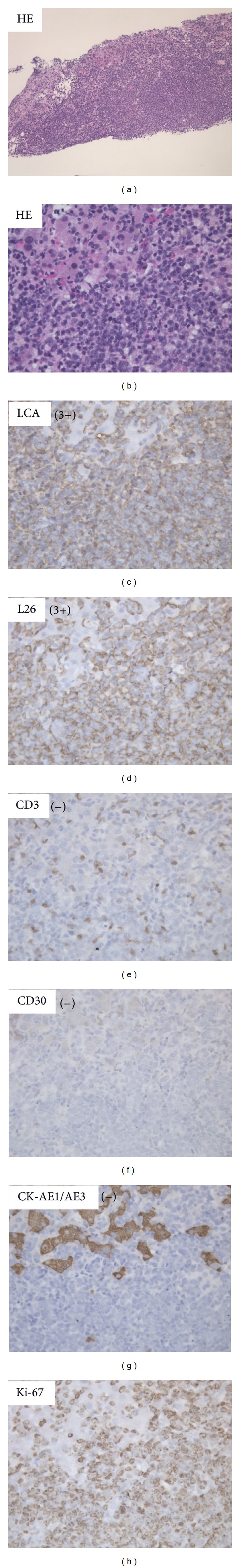
Histopathological findings. Histopathological findings from the biopsy specimens revealed the following. Diffuse and atypical large cells were revealed by hematoxylin and eosin staining ((a) and (b)). Immunohistochemical staining was positive for anti-LCA (CD45) and anti-L26 antibodies and negative for anti-CD3, anti-CD30, and anti-cytokeratin AE1/AE3 antibodies; Ki-67 staining was positive in 53% of cells ((c)–(h)).

**Figure 6:**
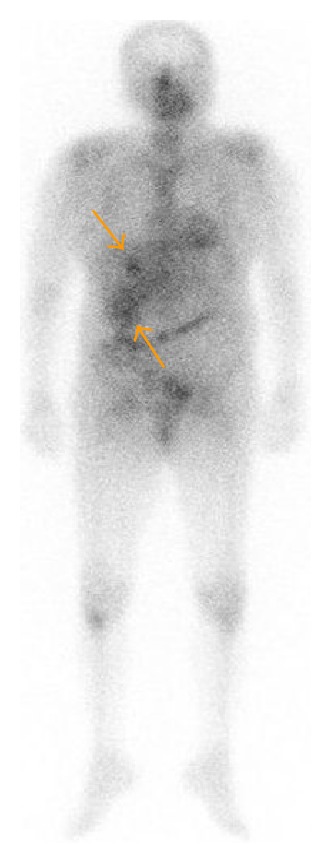
Gallium-67 scintigraphy. Two prominent hot spots were revealed in the liver.

An 82-year-old woman presented with a high-grade fever of 40°C and was admitted to our institution for intensive examination and treatment. The patient's medical history included type 2 diabetes mellitus diagnosed at 52 years, and oral hypoglycaemic agents were administered thereafter. She had no history of alcohol consumption or smoking. On admission, her consciousness level was alert; height, 145.6 cm; weight, 49.7 kg; body mass index, 23.4 kg/m^2^; blood pressure, 120/67 mmHg; heart rate, 81/min; respiratory rate, 14/min; body temperature, 38.0°C; saturated oxygen in arterial blood, 96% (room air). Mild anaemia was evident in her palpebral conjunctiva. Chest auscultation revealed no abnormal findings. The abdomen was soft and flat, with normal bowel sounds; slight tenderness was observed over the upper abdomen. Leg oedema was not evident. Chest radiography revealed a cardiothoracic ratio of 50.0%, without evidence of pulmonary congestion or pleural effusion. Abdominal radiography revealed no abnormal gas distribution. Noncontrast abdominal computed tomography (CT) revealed low-density masses at segments 5 and 8, suggestive of a liver abscess (Figures 1(a) and 1(b)). For further examinations, including contrast-enhanced abdominal CT, magnetic resonance imaging (MRI) and ultrasonography were scheduled. Contrast-enhanced abdominal CT showed a 30 × 30 mm mass with an enhanced margin at segment 8 in the arterial phase (Figures 2(a) and 2(b)). The contrast agents were washed out in the venous phase. In addition, a 63 × 52 mm mass with a density lower than that of liver parenchyma was observed at segment 8 in the portal phase (Figures 2(c) and 2(d)). On the basis of these findings, either a liver abscess or hepatocellular carcinoma was suspected. Noncontrast abdominal MRI revealed masses, 3.2 cm, 7 cm, and 2.2 cm in size, at segments 5, 6, and 8, respectively (Figures 3(a)–3(f)), which exhibited high signal intensity on T2- and diffusion-weighted imaging. Penetrating vessels were evident among these masses, suggestive of hepatic lymphoma (Figure 2(b)). Ultrasonography revealed low-echo masses, 3 cm, 6 cm, and 3 cm in size, at segments 5, 6, and 8, respectively (Figures 4(a)–4(d)). To confirm the diagnosis, a fine needle biopsy was scheduled. Histopathological analysis of the biopsied specimens showed diffuse and atypical large cells by hematoxylin and eosin staining (Figures 5(a) and 5(b)). Immunohistochemical staining was positive for anti-LCA (CD45) and anti-L26 antibodies and negative for anti-CD3, anti-CD30, and anti-cytokeratin AE1/AE3 antibodies; Ki-67 staining was positive in 53% of cells (Figures 5(c)–5(h)). On the basis of the imaging and liver biopsy results, a diagnosis of PHL, diffuse large B-cell lymphoma, was confirmed. Bone marrow biopsies revealed no evidence of tumour cells invasion. Serum soluble interleukin-2 receptor levels were markedly high (23,200 U/mL). Gallium-67 scintigraphy revealed 2 prominent hot spots in the liver (Figure 6). Chemotherapy was not indicated owing to the patient's age and poor performance status; thus, best supportive care was planned. On day 22 after admission, the patient died of pneumonia.

## 3. Discussion

PHL has been diagnosed in patients with an age of 8–78 years (average, 48 years) and predominantly occurs in men [1]. Its clinical symptoms include right upper abdominal pain (43%), weight loss (35%), and fever (22%) [1], and it has been associated with hepatitis B [2], hepatitis C [3], and human immunodeficiency viruses [4]. PHL patient positivity for the hepatitis B surface antigen and hepatitis C antibody is reportedly 22% and 58% of cases, respectively [5]. PHL affects the right lobe and bilateral lobes in 34.6% and 62.0% of cases, respectively [1]. The disease exhibits several morphologies: mononodular (71.2%), multinodular (25.0%), and diffuse (3.8%). Most PHL is derived from B-cell lymphoma (88.6%) and histologically diffuse large B-cell lymphoma (52.5%) [1].

The imaging findings for PHL comprise a low-density mass on noncontrast CT, a marginally or internally enhanced mass on contrast-enhanced CT, and a homogeneously low-echo mass on ultrasonography. It is impossible to confirm a PHL diagnosis by imaging alone as there are no characteristic imaging findings associated with this disease. While images of vessels penetrating tumours are suggestive of PHL, these vessels have a relatively low impact on the vascular structure of the liver. For a definite PHL diagnosis, histopathological examination of a fine needle biopsy is mandatory and has a reported accuracy of 85% [6]. The risk of tumour seeding by fine needle biopsy is low, at 0.005% [7], and, therefore, is recommended in cases where imaging results are suggestive of PHL.

PHL treatment options include resection of the affected lesion, a combination of chemotherapy and radiotherapy, and a combination of resection and chemotherapy, although no established treatment regimen exists. Niimi et al. [8] reported a case where histological examination of resected tumours after chemotherapy revealed no evidence of tumour cells. In general, PHL has a poor prognosis and the mean survival time following chemotherapy is 14 months. In contrast, the mean survival time following surgical treatment is 32.1 months [9], and localized PHL that can be completely resected has a relatively good prognosis. In patients with localized PHL, surgery is recommended as the first-line treatment.

## 4. Conclusion

We reported a case of PHL that was difficult to discriminate from a liver abscess. Imaging alone is insufficient to diagnose PHL; therefore, fine needle biopsy is recommended for a definitive diagnosis.
